# Comparison of Cervical Cytopathological Diagnosis Using Innovative Qi Brush and Traditional Cervex-Brush® Combi

**DOI:** 10.3389/fmed.2020.00369

**Published:** 2020-07-24

**Authors:** Yuliang Zou, Xiaoqian Tuo, Lei Wu, Yanli Liu, Xue Feng, Lanbo Zhao, Lu Han, Lei Wang, Yiran Wang, Huilian Hou, Guizhi Shi, Qiling Li

**Affiliations:** ^1^Department of Obstetrics and Gynecology, The First Affiliated Hospital of Xi'an Jiaotong University, Xi'an, China; ^2^Department of Obstetrics and Gynecology, Shaanxi Provincial People's Hospital, Xi'an, China; ^3^Department of Pathology, The First Affiliated Hospital of Xi'an Jiaotong University, Xi'an, China; ^4^Aviation General Hospital of Beijing, Medical University and Beijing Institute of Translational Medicine, University of Chinese Academy of Sciences, Beijing, China

**Keywords:** Qi brush, Cervex-Brush® Combi, cervical cancer, cervical cytology, screening

## Abstract

**Objectives:** To compare the effectiveness between Qi brush and Cervex-Brush® Combi for the diagnosis of cervical lesions.

**Methods:** After we registered a random-control clinical trial on the Chinese Clinical Trial Registry (No. XJTU1AF2017LSK-25), cervical cell samples were successively collected with both Qi brush and Cervex-Brush® Combi before undergoing colposcope. Colposcopy with biopsy was performed later. Histological diagnosis was regarded as the gold standard in this study. The following indices of the two brushes were compared: sampling degree of satisfaction and presence rate of metaplastic cells, together with sensitivity (Se), specificity (Sp), false positives (FP), false negatives (FN), positive predictive value (PPV), and negative predictive value (NPV). The kappa value was used to measure the inter-rater agreement of the Qi brush and Cervex-Brush® Combi in diagnosing cervical lesions.

**Results:** In total, 74 patients were enrolled in this study. The sensitivity, specificity, positive predictive value (PPV), and negative predictive value (NPV) of the Qi brush were 57.14, 86.84, 76.19, and 73.33%, respectively. For the Cervex-Brush® Combi, they were 26.92, 88.89, 63.63, and 62.75%, respectively. In addition, the Qi brush had a higher satisfied sampling rate (89.19%) than the Cervex-Brush® Combi (83.78%), and the *P*-value was 0.336 using Chi-square test. The kappa value was 0.444, which indicated a medium agreement between these two brushes, and the sensitivity of the Qi brush was higher than that of the Cervex-Brush® Combi, with significant statistical difference (*P* = 0.039<0.05).

**Conclusions:** The Qi brush was more effective than the Cervex-Brush® Combi for sampling and also had a slightly higher accuracy in diagnosing in cytology. In terms of social and economic benefits, the Qi brush may be a better cervical cytology collector.

## Introduction

Cervical cancer is the second-leading cause of cancer mortality among women aged 20–39 years worldwide, causing nine deaths per week in this age group, and the mortality among women in poor counties is twice that of women in affluent counties (1). In cervical cancer, squamous cell carcinoma is the most common type and accounts for almost 80% of cervical cancer. Squamous cell cancer generally begins with precancerous lesions, from low squamous intraepithelial lesions to high squamous intraepithelial lesions (2). Current cervical screening programs are used for detecting premalignant lesions to prevent invasive cervical cancer (3). Since the screening programs have been established, cervical cancer incidence rates have decreased by as much as 80% over the past 40 years in several Western countries (4, 5).

As screening with cervical cytology is considered to be an effective method for detecting cervical cancer and precancerous lesions, previous studies have illustrated that the sampling quality is associated with cervical sampling devices, which has resulted in around 60% false-negative results, especially with endogenous cervical cancer (6, 7). The collecting capacity of devices is largely affected by the shape of the device and its material, and a less-than-ideal device ultimately results in false results (8).

The sampling devices recommended by the European Guidelines for Quality Assurance are the combination of Cytobrush and Ayre spatula, the anatomical spatula, and the Cervex-Brush® Combi (9). A study of the Cervex-Brush Combi indicated that sampling with it resulted in a 2- to 3-fold harvest of endocervical cells when compared with the Cervex-Brush (10). Another study using the Cervex-Brush® Combi and Cytobrush + Ayres spatula, which included 1,235 patients, showed the specificities were 67.8 and 61.7%, respectively, and there was a better sensitivity for CIN2+ using Cervex-Brush® Combi when the cytology result was HSIL+ (6). However, the Cervex-Brush® Combi is relatively expensive for patients in developing countries.

**Figure 1:**
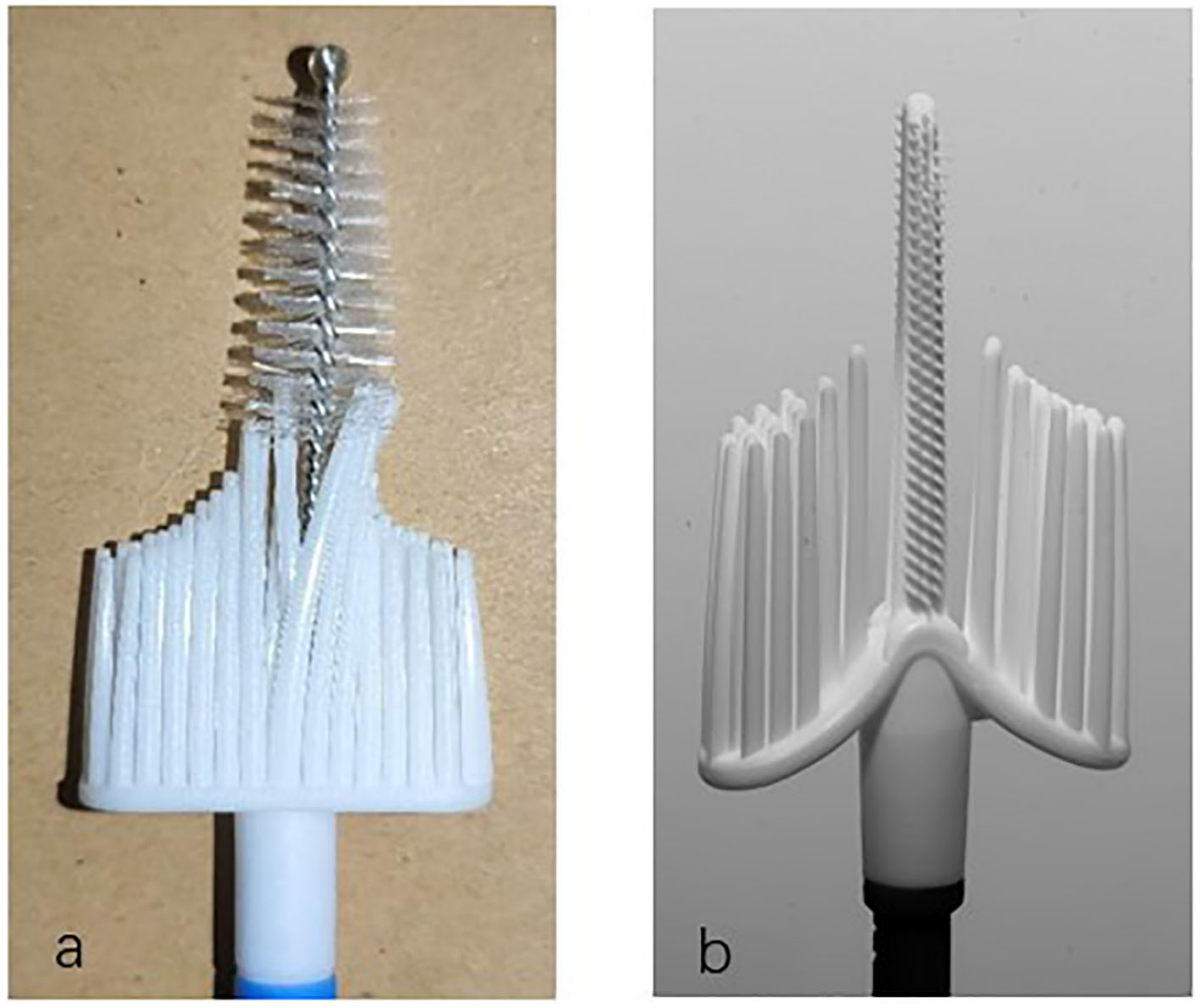
The Qi brush and the Cervex-Brush® Combi. **(a)** Qi brush. **(b)** Cervex-Brush® Combi.

Based on previous studies, our research group developed a patented cervical brush named the Qi brush (patent ZL 201420720356.8). The Qi brush (Figure 1) consists of endocervical and ectocervical arms with plastic bristles, sheath, and handle. The length of the endocervical arm is 2.0 cm, and the length of the bristles is 0.25–0.4 cm from top to near the ectocervical arm. The design of the endocervical arm allows the brush to fit the cervical canal and enable maximum sampling of cells. The ectocervical arm is semilunar and 1.0 cm long, and the length of the bristles is 1.5–2.5 cm from side to middle. As for the Cervex-Brush® Combi, the endocervical bristle is 1.0 cm long and is composed of short semicircular soft, flexible fibers, and its length is isometric. However, the length of cervical canal is 2.5–3.0 cm, which implies that an endocervical bristle of right length might result in more satisfied sampling.

In this study, we aimed to evaluate the accuracy of liquid-based cytology using the Qi brush vs. the Cervex-Brush® Combi for the diagnosis of cervical cytology.

## Materials and Methods

### Patients

From March 2018 to August 2019, a total of 74 patients were enrolled in this trial in the First Affiliated Hospital of Xi'an Jiao Tong University. The inclusion criterion of the study was as follows: patients who were detected with abnormal ThinPrep cytology test (TCT) results and who needed to undergo colposcopy with biopsy, the histological diagnosis being used as the gold standard. The exclusion criteria included total hysterectomy, cervical surgery including conization, loop electrosurgical excision procedure (LEEP), radiation therapy, acute inflammation, and confirmation of pregnancy. The study was approved by the Institutional Review Board of the First Affiliated Hospital of Xi'an Jiaotong University. Informed consent was obtained from all patients.

### Sample Collection

All participants were numbered according to the registration order. Before the colposcopy, the patients in the odd-numbered group were sampled with the Qi brush (Xi'an Meijiajia Bio-Technologies Co. Ltd., China) (Figure 1a) first and then the Cervex-Brush® Combi (Rovers® Medical Devices, Netherlands, 89171-022) (Figure 1b) (6), while the patients in the even-numbered group were sampled in the reverse order. The instructions for use of these two brushes were as follows: expose the cervix, wipe off the secretion with a cotton swab, gingerly insert the brush into the endocervical canal until the lateral bristles touch the ectocervix, rotate t turns clockwise, and finally insert the brush head into the preservation solution bottle (6). The two bottles were labeled clearly and only the operating doctor knew the tags and their corresponding variables. Finally, the samples were delivered to the Department of Pathology for further staining procedures and cytological diagnosis. Participants then underwent colposcopy with biopsy after collection of cytological samples.

### Cytological and Histological Diagnosis

Both cytological and histological diagnoses were made by two experienced pathologists using conventional optical microscopy. The pathologists evaluated the quantity of columnar cells, squamous cells, and metaplastic squamous cells from both endo- and exocervical samples. Samples containing one cellular group with >15 cells were defined as poor samples. In other words, the presence of two or more cellular groups of at least 15 cells was regarded as acceptable (11).

The Bethesda 3-tier system was used as the cervical cytological diagnostic criteria. And positive results included: (1) atypical squamous cells of unknown significance (ASC-US); (2) lesion that cannot exclude high-grade squamous intraepithelial lesion (ASC-H); (3) low-grade squamous intraepithelial lesion (LSIL); (4) low-grade squamous intraepithelial lesion (LSIL); (5) high-grade squamous intraepithelial lesion (HSIL); (6) squamous cell carcinoma (SCC) (12). As for the histological diagnosis, CIN1-3 (Cervical Intraepithelial Neoplasia) and squamous carcinoma were regarded as positive results according to the WHO classification (13).

### Statistical Analysis

The data of all the participants were obtained from their medical records. In this study, we compared the sampling degree of satisfaction and presence rate of metaplastic cells by using the Qi brush and the Cervex-Brush® Combi. Sensitivity (Se), specificity (Sp), false positives (FP), false negatives (FN), positive predictive values (PPV), and negative predictive values (NPV) were analyzed for the comparison of the diagnostic accuracy of the two brushes. Chi-square test was used for numerical variables, and *P* < 0.05 indicated significant statistical difference. The inter-rater agreement of the Qi brush and the Cervex-Brush® Combi in diagnosing cervical lesions was measured by Cohen's kappa coefficient. Analysis was processed by SPSS22.0 statistical software.

## Results

### Characterization of Patients Included

Overall, 74 patients from the Inpatient Department of the First Affiliated Hospital of Xi'an Jiao Tong University were enrolled in this study, and all of the participants ultimately underwent colposcopy and biopsy. The characteristics of the patients are shown in Table 1. The age of the patients included ranged from 21 to 68 years, with an average age of 45.39 years. Most of the patients were between 30 and 64 years of age (*n* = 62,83.78%), which was the age recommended for screening. As for the choice of contraceptive method, oral contraceptives (*n* = 5, 6.76%) were the least common choice, while IUD (intrauterine device) (*n* = 18, 24.32%) and condoms (*n* = 17, 22.97%) were more commonly used. Since 27 of the patients were menopausal women and 9 of them were at perimenopausal stage, natural menopause and sterilization were the most common choices. Among the patients enrolled, 54 of them were infected with HrHPV (high-risk papilloma virus), and 32 of them were positive for HPV16, HPV18, or both.

**Table 1:** Characteristics of the study population.

**Variables**	**Category**	***n* (%)**
Age	<30 years	5 (6.76%)
	30–64 years	62 (83.78%)
	>64 years	7 (9.46%)
Contraceptive methods	Oral contraceptive	5 (6.76%)
	Intrauterine device (IUD)	18 (24.32%)
	Condom	17 (22.97%)
	Others (menopause, sterilization, etc.)	34 (45.95%)
HPV infection	Without HPV infection	14 (18.92%)
	HrHPV (16.18) infection	32 (43.24%)
	HrHPV (others) infection	22^*^ (29.73%)
	No results	6 (8.11%)
Menstrual period	Luteal phase	27 (36.49%)
	Follicular phase	11 (14.86%)
	Menopause	27 (36.49%)
	Perimenopause period	9 (12.16%)

*HrHPV, High-risk human papilloma virus*.

22* *: Among the participants, one patient had the positive HrHPV result without stating specific type, and was listed in this group*.

### Evaluation of Sampling Degree of Satisfaction

There were eight poor samples using the Qi brush and 12 poor samples using the Cervex-Brush® Combi. Among the eight samples using the Qi brush, five were sampled after using the Cervex-Brush® Combi. As for the 12 samples using the Cervex-Brush® Combi, seven were sampled after using the Qi brush. In addition, two patients' samples were poor samples no matter which brush was used for sampling. Ultimately, the satisfied sampling rate of the Qi brush and the Cervex-Brush® Combi were 89.19 and 83.78%, respectively. However, *P* > 0.05 (*P* = 0.336) using Chi-square test indicated there was no statistical difference using those two brushes.

As for obtaining squamous metaplastic cells using different instruments, there were 46/66 samples (69.7%) and 44/62 samples (70.97%) that used the Qi brush and the Cervex-Brush® Combi, respectively. *P* > 0.05 (*P* = 0.875) using Chi-square test indicated there was no statistical difference using those two brushes in obtaining metaplastic cells.

### Cytological and Histological Diagnosis

Tables 2, 3 show the comparison of cytological and histological diagnoses and the diagnostic accuracy between the Qi brush and the Cervex-Brush® Combi, respectively. The diagnostic sensitivity of the Qi brush and the Cervex-Brush® Combi was 57.14 and 26.92%, respectively, with significant statistical difference (*P* = 0.039 <0.05). The specificity was 86.84 and 88.89%, respectively. The Qi brush had a diagnostic accuracy in PPV of 76.19% and in NPV of 73.33%, while the Cervex-Brush® Combi was 63.63 and 62.75%, respectively. The comparison of cytological diagnosis between the two brushes is represented in Table 4. The kappa value was 0.444, which indicates a medium agreement between these two brushes, and the diagnostic sensitivity of the Qi brush was significantly higher than that of the Cervex-Brush® Combi.

**Table 2:** Comparison of histology and cytology diagnosis by Qi Brush and Combi Brush.

		**Histology**		**Total**
		**Positive**	**Negative**	
Cytology by Qi Brush	Positive	16	5	21
	Negative	12	33	45
	Total	28	38	66
Cytology by Combi Brush	Positive	7	4	11
	Negative	19	32	51
	Total	26	36	62

**Table 3:** Diagnostic accuracy of Qi Brush and combi Brush.

	**Se (%)**	**Sp (%)**	**FN (%)**	**FP (%)**	**PPV (%)**	**NPV (%)**
Qi Brush	57.14	86.84	42.86	13.16	76.19	73.33
combi brush	26.92	88.89	73.08	11.11	63.63	62.75

*Se, sensitivity; Sp, specificity; FN, false negative; FP, false positive; PPV, positive prediction value; NPV, negative prediction value*.

**Table 4:** Comparison of cytological diagnosis between Qi brush and combi brush.

		**Combi Brush**						**Total**
		**NILM**	**ASCUS**	**ASC-H**	**LSIL**	**HSIL**	**SCC**	
Qi Brush	NILM	35	2	0	0	0	0	45
	ASCUS	7	2	1	0	1	0	11
	ASC-H	1	1	1	0	0	0	3
	LSIL	1	1	0	0	1	0	3
	HSIL	1	0	0	0	0	0	1
	SCC	0	0	0	0	0	0	0
	Total	46	6	2	0	2	0	56

## Discussion

Currently, there are two types of screening terms for cervical cancer, the Papanicolaou test and the HPV test. As for cytology screening, since liquid-based cytology was introduced in the 1990s, it has seemed to be a better tool for processing cervical samples (14–16). Sampling devices recommended by the European Guidelines for Quality Assurance include the cervical brush (Cervex-Brush, Rovers), a combination of a spatula for ectocervical sampling and an endocervical brush for endocervical sampling, and an extended-tip spatula alone (9). The combination of the Cytobrush and the Ayre spatula was once the most commonly used sampling device for the reason that the Ayre spatula alone failed to collect cells in the cervical canal. However, the use of two sampling devices generated more blood contamination on the background of the slides (17). The Aylesbury spatula was the most commonly used extended-tip spatula used alone and was reported to obtain more endocervical cells than other spatulas (18). Since the spatula can't cannot be applied to patients with cervical stenosis, the Cervex-Brush—which had the advantages of painless operation, low bleeding volume in patients, and the sampling of cervical cells—was later more commonly used. The Cervex-Brush® Combi used in this research combined the important features of the original Cervex-Brush® with the benefits of the EndoCervex-Brush® and resulted in a 2- to 3-fold increase in the number of sampled endocervical cells compared to the Cervex-Brush (10). As depicted in the product brochure and Figure 1b, the endocervical bristle of the Cervex-Brush® Combi is 1.0 cm long, it is composed of short semicircular soft, flexible fibers, and its length is isometric. Comparatively, the endocervical bristle of the Qi brush is 2.0 cm long and is spirally formed with short burrs, which gradually become longer and denser from top to bottom according to the anatomical structure of the cervical canal.

Although in theory the design of the Qi brush could lead to higher sampling satisfaction rate and more chances for obtaining metaplastic cells, the results in this study showed there is no statistical difference between the two brushes. Moreover, the sampling satisfaction rate of the Qi brush and the Cervex-Brush® Combi is 89.19 and 83.78%, respectively, which is lower than De Palo's 94.68% and Abdali's 92.1% (19, 20). This might be because the two types of cervical brushes were sampled alternately. When one of the cervical brushes was sampled first and rotated 2–3 turns clockwise, there was a risk of bleeding caused by cervical columnar epithelial injury. Subsequently, the background of the cervical slides was contaminated with red blood cells, resulting in decreased sampling satisfaction rate.

As Woodman and Mitchell noted, it is actually the presence of metaplastic cells that predicts squamous dysplasia, and lumping the columnar cells and metaplastic cells together weakens the association (21, 22). In this research, the metaplastic cells presence rate of the Qi brush and the Cervex-Brush® Combi is 69.7 and 70.97% (*P* = 0.875>0.05), respectively, with no significant difference between them. It could also result in low sensitivity and false-negative results, in addition to the sampling satisfaction rate.

In this study, taking the histological results as the gold standard, we compared the diagnostic accuracy using the Qi brush and the Cervex-Brush® Combi. Compared with the Cervex-Brush® Combi, the Qi brush had a higher sensitivity (57.14%) and PPV (76.19%). The specificity and NPV of the Qi brush were 86.84 and 73.33%, respectively, while those of the Cervex-Brush® Combi were 88.89 and 62.75%. However, the sensitivity of the Cervex-Brush® Combi in this study was lower than in the previous study, which might have been due to the professionalism of the operators and the relatively small number of patients enrolled in this study (6, 10, 23, 24). In addition, the price of the Qi brush is less than that of the Cervex-Brush® Combi, which is around 20 yuan. That makes the Qi brush an economical and practical choice for screening when combined with the economic social benefits.

The limitations of this study are due to the small number of participants and the failure to strictly control operational normatives. Consequently, further study based on larger study populations is under way to verify the use of the Qi brush in cervical cytology sampling.

Our study showed that cervical cytology sampled with the Qi brush has a higher accuracy with histological diagnosis in detecting cervical cytological results. In addition, the Qi brush has a lower insufficient sampling rate. After the social and economic benefits are factored in, the Qi brush may be a better cervical cytology collector.

## Data Availability Statement

The raw data supporting the conclusions of this article will be made available by the authors without undue reservation, to any qualified researcher.

## Ethics Statement

The studies involving human participants were reviewed and approved by the Institutional Review Board of the First Affiliated Hospital of Xi'an Jiaotong University. The patients/participants provided their written informed consent to participate in this study.

## Author Contributions

QL, YZ, and XT devised the conceptual idea. LWu, YL, XF, LZ, HH, and GS executed the work. XT wrote the manuscript. LH, LWa, and YW critically reviewed the manuscript. All authors contributed to the article and approved the submitted version.

## Conflict of Interest

The authors declare that the research was conducted in the absence of any commercial or financial relationships that could be construed as a potential conflict of interest.
